# 2-Amino-5-methyl­pyridinium picolinate 0.63-hydrate

**DOI:** 10.1107/S1600536810018180

**Published:** 2010-05-22

**Authors:** Madhukar Hemamalini, Hoong-Kun Fun

**Affiliations:** a X-ray Crystallography Unit, School of Physics, Universiti Sains Malaysia, 11800 USM, Penang, Malaysia

## Abstract

The asymmetric unit of the title compound, C_6_H_9_N_2_^+^·C_6_H_4_NO_2_^−^·0.63H_2_O, contains two crystallographically independent 2-amino-5-methyl­pyridinium cations, a pair of picolinate anions and two water mol­ecules, one with an occupancy of 0.25. Both the 2-amino-5-methyl­pyridine mol­ecules are protonated at the pyridine N atoms. In the crystal structure, the cations, anions and water mol­ecules are linked *via* N—H⋯O, N—H⋯N and O—H⋯O hydrogen bonds, as well as by C—H⋯O contacts, forming a chain along the *b* axis. In addition, weak π–π inter­actions are observed between pyridinium rings, with centroid–centroid distances of 3.5306 (13) Å.

## Related literature

For background to the chemistry of substituted pyridines, see: Pozharski *et al.* (1997 ▶); Katritzky *et al.* (1996 ▶); Navarro Ranninger *et al.* (1985 ▶); Luque *et al.* (1997 ▶); Qin *et al.* (1999 ▶); Yip *et al.* (1999 ▶); Ren *et al.* (2002 ▶); Rivas *et al.* (2003 ▶); Jin *et al.* (2001 ▶); Albrecht *et al.* (2003 ▶); Nahringbauer & Kvick (1977 ▶). For details of hydrogen bonding, see: Jeffrey & Saenger (1991 ▶); Jeffrey (1997 ▶); Scheiner (1997 ▶). For details of picolinic acid, see: Mahler & Cordes (1971 ▶); Ogata *et al.* (2000 ▶). For hydrogen-bond motifs, see: Bernstein *et al.* (1995 ▶). For bond-length data, see: Allen *et al.* (1987 ▶). For the stability of the temperature controller used in the data collection, see: Cosier & Glazer (1986 ▶).
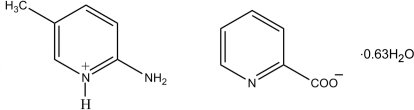


## Experimental

### 

#### Crystal data

C_6_H_9_N_2_^+^·C_6_H_4_NO_2_^−^·0.63H_2_O
*M* *_r_* = 242.51
Orthorhombic, 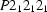

*a* = 12.126 (3) Å
*b* = 13.842 (3) Å
*c* = 14.318 (3) Å
*V* = 2403.4 (10) Å^3^
*Z* = 8
Mo *K*α radiation
μ = 0.10 mm^−1^
*T* = 100 K
0.28 × 0.20 × 0.09 mm

#### Data collection

Bruker APEXII DUO CCD area-detector diffractometer
Absorption correction: multi-scan (*SADABS*; Bruker, 2009 ▶) *T*_min_ = 0.973, *T*_max_ = 0.991
50363 measured reflections
3955 independent reflections
3119 reflections with *I* > 2σ(*I*)
*R*_int_ = 0.068

#### Refinement

*R*[*F* ^2^ > 2σ(*F* ^2^)] = 0.046
*wR*(*F* ^2^) = 0.141
*S* = 1.09
3955 reflections
353 parameters
H atoms treated by a mixture of independent and constrained refinement
Δρ_max_ = 0.33 e Å^−3^
Δρ_min_ = −0.35 e Å^−3^

### 

Data collection: *APEX2* (Bruker, 2009 ▶); cell refinement: *SAINT* (Bruker, 2009 ▶); data reduction: *SAINT*; program(s) used to solve structure: *SHELXTL* (Sheldrick, 2008 ▶); program(s) used to refine structure: *SHELXTL*; molecular graphics: *SHELXTL*; software used to prepare material for publication: *SHELXTL* and *PLATON* (Spek, 2009 ▶).

## Supplementary Material

Crystal structure: contains datablocks global, I. DOI: 10.1107/S1600536810018180/sj5005sup1.cif

Structure factors: contains datablocks I. DOI: 10.1107/S1600536810018180/sj5005Isup2.hkl

Additional supplementary materials:  crystallographic information; 3D view; checkCIF report

## Figures and Tables

**Table 1:** Hydrogen-bond geometry (Å, °)

*D*—H⋯*A*	*D*—H	H⋯*A*	*D*⋯*A*	*D*—H⋯*A*
O2*W*—H2*W*2⋯O2*A*^i^	0.82	2.00	2.812 (2)	170
N1*A*—H1*NA*⋯O2*B*^ii^	0.99 (2)	1.69 (2)	2.669 (2)	170 (2)
N2*A*—H2*NA*⋯O1*B*^ii^	0.94 (3)	1.89 (3)	2.829 (2)	178 (3)
N2*A*—H3*NA*⋯N3*A*	0.94 (3)	2.08 (3)	3.019 (3)	177 (2)
N1*B*—H1*NB*⋯O2*A*^i^	0.96 (3)	1.68 (3)	2.642 (2)	173 (3)
N2*B*—H2*NB*⋯N3*B*	0.83 (3)	2.22 (3)	3.040 (2)	173 (2)
N2*B*—H3*NB*⋯O1*A*^i^	0.93 (2)	1.90 (2)	2.831 (3)	175 (2)
C5*A*—H5*AA*⋯O2*W*^iii^	0.93	2.39	3.319 (3)	175
C7*A*—H7*AA*⋯O2*B*^iv^	0.93	2.42	3.217 (3)	144
C8*A*—H8*AA*⋯O2*W*^v^	0.93	2.50	3.339 (3)	151

Symmetry codes: (i) 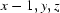; (ii) 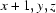; (iii) 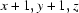; (iv) 
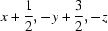
; (v) 
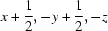
.

**Fig. 1.**
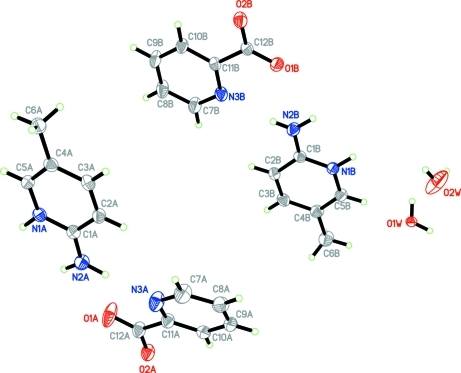
The asymmetric unit of the title compound. Displacement ellipsoids are drawn at the 50% probability level.

**Fig. 2.**
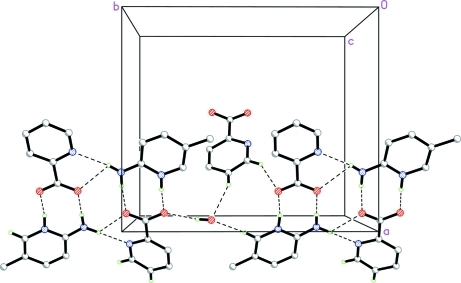
The crystal packing of the title compound, showing hydrogen-bonded (dashed lines) networks. H atoms not involved in hydrogen bond interactions are omitted for clarity.

## supplementary crystallographic information

### Comment

Pyridine and its derivatives play an important role in heterocyclic chemistry (Pozharski *et al.*, 1997; Katritzky *et al.*, 1996). They are often involved in hydrogen-bond interactions (Jeffrey & Saenger, 1991; Jeffrey, 1997; Scheiner, 1997). There are numerous examples of 2-amino-substituted pyridine compounds in which the 2-aminopyridines act as neutral ligands (Navarro Ranninger *et al.*, 1985; Luque *et al.*, 1997; Qin *et al.*, 1999; Yip *et al.*, 1999; Ren *et al.*, 2002; Rivas *et al.*, 2003) or as protonated cations (Luque *et al.*, 1997; Jin *et al.*, 2001; Albrecht *et al.*, 2003).  Picolinic acid (pyridine-2-carboxylic acid) is a well known terminal tryptophan metabolite (Mahler & Cordes, 1971). It induces apoptosis in leukaemia HL-60 cells (Ogata *et al.*, 2000). Since our aim is to study some interesting hydrogen bonding interactions, the crystal structure of the title compound is presented here.

The asymmetric unit of the title compound consists of two crystallographically independent 2-amino-5-methylpyridinium cations (A and B), two picolinate anions (A and B) and two water molecules, O1W and O2W (with occupancies 0.25 and 1.0, respectively), (Fig. 1). Each 2-amino-5-methylpyridinium cation is planar, with a maximum deviation of 0.024 (2) Å for atom C6A in cation A and  0.005 (2) Å for atom C1B in cation B. In the cations, protonation at atoms N1A and N1B lead to a slight increase in the C1A—N1A—C5A [123.2 (2)°] and C1B—N1B—C5B [123.0 (2)°] angles compared to those observed in an unprotonated structure (Nahringbauer & Kvick, 1977). The bond lengths (Allen *et al.*, 1987) and angles are normal.

In the crystal structure (Fig. 2), the carboxylate groups of each picolinate anion interact with the corresponding 2-amino-5-methylpyridinium cations via a pair of N—H···O hydrogen bonds forming an *R*^2^_2_(8)  ring motif (Bernstein *et al.*, 1995). The ionic units are linked by N—H···N, N—H···O, O—H···O and C—H···O (Table 1) hydrogen bonds, forming a one-dimensional chain along the *b*-axis.  The crystal structure is further stabilized by π–π interactions involving the pyridinium (N1A/C1A–C5A) and pyridinium (N1B/C1B–C5B) rings, with centroid-to-centroid distance of 3.5306 (13) Å [symmetry code: 1-x, 1/2+y, 1/2-z].

### Experimental

Hot methanol solutions (20 ml) of 2-amino-5-methylpyridine (54 mg, Aldrich) and picolinic acid (62 mg, Merck) were mixed and warmed over a a magnetic stirrer hotplate for a few minutes. The resulting solution was allowed to cool slowly at room temperature and crystals of the title compound appeared after a few days.

### Refinement

Atoms H1NA, H2NA, H3NA, H1NB, H2NB and H3NB were located from a difference Fourier map and freely refined. The remaining hydrogen atoms were positioned geometrically [C–H = 0.93 Å, N–H = 0.82 (3)–0.97 (4) Å and O–H = 0.8098–0.8226 Å] and were refined using a riding model, with U_iso_(H) = 1.2 U_eq_(C, N) or 1.5 U_eq_(O). The methyl H atoms were positioned geometrically and were refined using a riding model, with U_iso_(H) = 1.5U_eq_(C). A rotating group model was used for the methyl group. The occupancy of the (O1W) water molecule was initially refined and then fixed at 25% occupancy in the final refinement. In the absence of significant anomalous scattering effects, 3139 Friedel pairs were merged.

### Crystal data

**Table:**

C_6_H_9_N_2_^+^·C_6_H_4_NO_2_^−^·0.63H_2_O	*F*(000) = 1026
*M**_r_* = 242.51	*D*_x_ = 1.340 Mg m^−^^3^
Orthorhombic, *P*2_1_2_1_2_1_	Mo *K*α radiation, λ = 0.71073 Å
Hall symbol: P 2ac 2ab	Cell parameters from 7433 reflections
*a* = 12.126 (3) Å	θ = 2.2–29.8°
*b* = 13.842 (3) Å	µ = 0.10 mm^−^^1^
*c* = 14.318 (3) Å	*T* = 100 K
*V* = 2403.4 (10) Å^3^	Block, colourless
*Z* = 8	0.28 × 0.20 × 0.09 mm

### Data collection

**Table:**

Bruker APEXII DUO CCD area-detector diffractometer	3955 independent reflections
Radiation source: fine-focus sealed tube	3119 reflections with *I* > 2σ(*I*)
graphite	*R*_int_ = 0.068
φ and ω scans	θ_max_ = 30.2°, θ_min_ = 2.2°
Absorption correction: multi-scan (*SADABS*; Bruker, 2009)	*h* = −17→17
*T*_min_ = 0.973, *T*_max_ = 0.991	*k* = −19→19
50363 measured reflections	*l* = −20→20

### Refinement

**Table:**

Refinement on *F*^2^	Primary atom site location: structure-invariant direct methods
Least-squares matrix: full	Secondary atom site location: difference Fourier map
*R*[*F*^2^ > 2σ(*F*^2^)] = 0.046	Hydrogen site location: inferred from neighbouring sites
*wR*(*F*^2^) = 0.141	H atoms treated by a mixture of independent and constrained refinement
*S* = 1.09	*w* = 1/[σ^2^(*F*_o_^2^) + (0.0779*P*)^2^ + 0.4137*P*] where *P* = (*F*_o_^2^ + 2*F*_c_^2^)/3
3955 reflections	(Δ/σ)_max_ = 0.001
353 parameters	Δρ_max_ = 0.33 e Å^−^^3^
0 restraints	Δρ_min_ = −0.35 e Å^−^^3^

### Special details

**Table:**

Experimental. The crystal was placed in the cold stream of an Oxford Cryosystems Cobra open-flow nitrogen cryostat (Cosier & Glazer, 1986) operating at 100.0 (1) K.
Geometry. All s.u.'s (except the s.u. in the dihedral angle between two l.s. planes) are estimated using the full covariance matrix. The cell s.u.'s are taken into account individually in the estimation of s.u.'s in distances, angles and torsion angles; correlations between s.u.'s in cell parameters are only used when they are defined by crystal symmetry. An approximate (isotropic) treatment of cell s.u.'s is used for estimating s.u.'s involving l.s. planes.
Refinement. Refinement of F^2^ against ALL reflections. The weighted R-factor wR and goodness of fit S are based on F^2^, conventional R-factors R are based on F, with F set to zero for negative F^2^. The threshold expression of F^2^ > 2σ(F^2^) is used only for calculating R-factors(gt) etc. and is not relevant to the choice of reflections for refinement. R-factors based on F^2^ are statistically about twice as large as those based on F, and R- factors based on ALL data will be even larger.

### Fractional atomic coordinates and isotropic or equivalent isotropic displacement parameters (Å^2^)

**Table:**

	*x*	*y*	*z*	*U*_iso_*/*U*_eq_	Occ. (<1)
N1A	0.97657 (16)	0.86544 (15)	0.09594 (13)	0.0235 (4)	
N2A	0.98590 (18)	0.69933 (17)	0.08274 (15)	0.0291 (5)	
C1A	0.93479 (19)	0.78196 (18)	0.06311 (15)	0.0231 (4)	
C2A	0.83756 (19)	0.78782 (18)	0.00781 (17)	0.0253 (5)	
H2AA	0.8060	0.7319	−0.0162	0.030*	
C3A	0.79046 (19)	0.87571 (18)	−0.01001 (17)	0.0257 (5)	
H3AA	0.7273	0.8789	−0.0467	0.031*	
C4A	0.83615 (19)	0.96169 (17)	0.02636 (17)	0.0247 (5)	
C5A	0.92944 (19)	0.95332 (17)	0.07947 (16)	0.0238 (5)	
H5AA	0.9613	1.0085	0.1048	0.029*	
C6A	0.7824 (2)	1.05808 (19)	0.01044 (19)	0.0306 (5)	
H6AA	0.8170	1.1058	0.0493	0.046*	
H6AB	0.7055	1.0539	0.0258	0.046*	
H6AC	0.7904	1.0761	−0.0539	0.046*	
O1A	1.0576 (2)	0.51193 (14)	0.1466 (2)	0.0585 (8)	
O2A	1.06051 (16)	0.35162 (13)	0.15326 (14)	0.0349 (4)	
N3A	0.8965 (2)	0.50822 (17)	0.01507 (19)	0.0407 (6)	
C7A	0.8132 (2)	0.5040 (2)	−0.0465 (2)	0.0425 (7)	
H7AA	0.7946	0.5602	−0.0785	0.051*	
C8A	0.7533 (2)	0.4213 (2)	−0.0653 (2)	0.0373 (6)	
H8AA	0.6959	0.4219	−0.1084	0.045*	
C9A	0.7809 (2)	0.3379 (2)	−0.0184 (2)	0.0332 (6)	
H9AA	0.7420	0.2811	−0.0288	0.040*	
C10A	0.8683 (2)	0.34009 (19)	0.04496 (18)	0.0280 (5)	
H10A	0.8890	0.2846	0.0771	0.034*	
C11A	0.9239 (2)	0.42690 (18)	0.05917 (18)	0.0282 (5)	
C12A	1.0218 (2)	0.43223 (19)	0.1257 (2)	0.0319 (5)	
N1B	0.24603 (16)	0.35896 (15)	0.24965 (14)	0.0233 (4)	
N2B	0.25938 (19)	0.52545 (16)	0.24544 (15)	0.0271 (4)	
C1B	0.29982 (19)	0.44099 (17)	0.27328 (16)	0.0230 (4)	
C2B	0.39853 (18)	0.43069 (17)	0.32614 (17)	0.0251 (5)	
H2BA	0.4384	0.4851	0.3438	0.030*	
C3B	0.4344 (2)	0.34168 (19)	0.35063 (18)	0.0280 (5)	
H3BA	0.4989	0.3362	0.3853	0.034*	
C4B	0.3767 (2)	0.25626 (18)	0.32509 (17)	0.0276 (5)	
C5B	0.2821 (2)	0.26944 (17)	0.27415 (17)	0.0251 (5)	
H5BA	0.2415	0.2157	0.2559	0.030*	
C6B	0.4152 (3)	0.15790 (19)	0.3523 (2)	0.0392 (7)	
H6BA	0.3732	0.1102	0.3191	0.059*	
H6BB	0.4052	0.1491	0.4182	0.059*	
H6BC	0.4919	0.1511	0.3371	0.059*	
O1B	0.16897 (15)	0.71003 (12)	0.20473 (13)	0.0300 (4)	
O2B	0.16686 (15)	0.87068 (13)	0.18961 (13)	0.0310 (4)	
N3B	0.35568 (17)	0.71727 (15)	0.30874 (15)	0.0279 (4)	
C7B	0.4429 (2)	0.72461 (19)	0.3658 (2)	0.0358 (6)	
H7BA	0.4767	0.6679	0.3857	0.043*	
C8B	0.4858 (2)	0.8121 (2)	0.3969 (2)	0.0373 (6)	
H8BA	0.5458	0.8134	0.4373	0.045*	
C9B	0.4378 (2)	0.89624 (19)	0.36688 (19)	0.0332 (6)	
H9BA	0.4650	0.9558	0.3860	0.040*	
C10B	0.3477 (2)	0.89054 (18)	0.30724 (17)	0.0271 (5)	
H10B	0.3142	0.9465	0.2851	0.033*	
C11B	0.30822 (18)	0.80070 (17)	0.28102 (16)	0.0227 (4)	
C12B	0.20592 (18)	0.79216 (17)	0.21971 (16)	0.0222 (4)	
O1W	0.1654 (5)	0.0578 (4)	0.2767 (5)	0.0227 (12)	0.25
H1W1	0.1196	0.0966	0.2937	0.034*	0.25
H2W1	0.1398	0.0069	0.2582	0.034*	0.25
O2W	0.0279 (3)	0.15220 (18)	0.17835 (19)	0.0758 (10)	
H1W2	−0.0219	0.1402	0.2150	0.114*	
H2W2	0.0312	0.2103	0.1667	0.114*	
H1NA	1.044 (3)	0.858 (3)	0.131 (3)	0.050 (10)*	
H2NA	1.049 (3)	0.702 (3)	0.124 (3)	0.060 (11)*	
H3NA	0.959 (3)	0.638 (2)	0.064 (2)	0.034 (8)*	
H1NB	0.177 (3)	0.359 (3)	0.219 (3)	0.061 (11)*	
H2NB	0.292 (3)	0.574 (2)	0.263 (2)	0.037 (9)*	
H3NB	0.193 (3)	0.525 (3)	0.212 (2)	0.045 (9)*	

### Atomic displacement parameters (Å^2^)

**Table:**

	*U*^11^	*U*^22^	*U*^33^	*U*^12^	*U*^13^	*U*^23^
N1A	0.0208 (9)	0.0264 (10)	0.0232 (9)	0.0005 (8)	−0.0022 (8)	0.0015 (8)
N2A	0.0295 (10)	0.0258 (11)	0.0318 (11)	0.0013 (9)	−0.0059 (9)	0.0014 (9)
C1A	0.0233 (10)	0.0254 (11)	0.0206 (10)	0.0000 (9)	0.0007 (9)	0.0010 (9)
C2A	0.0242 (10)	0.0245 (11)	0.0274 (11)	−0.0028 (9)	−0.0029 (9)	−0.0013 (9)
C3A	0.0205 (10)	0.0314 (12)	0.0253 (11)	−0.0022 (9)	−0.0019 (9)	0.0026 (9)
C4A	0.0231 (10)	0.0248 (11)	0.0263 (11)	0.0013 (9)	0.0032 (9)	0.0031 (9)
C5A	0.0240 (10)	0.0217 (11)	0.0257 (11)	−0.0007 (9)	0.0002 (9)	−0.0008 (9)
C6A	0.0275 (11)	0.0281 (12)	0.0361 (13)	0.0001 (10)	−0.0007 (10)	0.0047 (11)
O1A	0.0586 (14)	0.0248 (10)	0.0922 (19)	−0.0069 (10)	−0.0532 (14)	0.0083 (11)
O2A	0.0308 (9)	0.0263 (9)	0.0475 (11)	−0.0005 (7)	−0.0147 (8)	0.0024 (8)
N3A	0.0420 (13)	0.0305 (12)	0.0495 (14)	−0.0023 (10)	−0.0252 (12)	0.0033 (11)
C7A	0.0435 (16)	0.0355 (15)	0.0483 (16)	0.0052 (13)	−0.0225 (14)	0.0034 (13)
C8A	0.0311 (13)	0.0435 (16)	0.0373 (14)	0.0067 (12)	−0.0122 (11)	−0.0120 (12)
C9A	0.0267 (12)	0.0338 (14)	0.0392 (14)	−0.0009 (10)	−0.0045 (11)	−0.0136 (11)
C10A	0.0242 (11)	0.0293 (12)	0.0305 (12)	0.0010 (10)	0.0006 (9)	−0.0032 (10)
C11A	0.0265 (11)	0.0263 (12)	0.0318 (12)	−0.0001 (10)	−0.0057 (10)	−0.0009 (10)
C12A	0.0298 (12)	0.0253 (12)	0.0407 (14)	−0.0018 (10)	−0.0125 (11)	0.0017 (11)
N1B	0.0228 (9)	0.0216 (10)	0.0255 (9)	0.0017 (8)	−0.0028 (8)	−0.0009 (8)
N2B	0.0292 (10)	0.0215 (10)	0.0305 (11)	0.0005 (9)	−0.0057 (9)	0.0004 (8)
C1B	0.0225 (10)	0.0235 (11)	0.0230 (10)	0.0006 (9)	0.0019 (8)	0.0003 (9)
C2B	0.0209 (10)	0.0249 (11)	0.0294 (12)	−0.0010 (9)	−0.0036 (9)	−0.0018 (9)
C3B	0.0246 (11)	0.0298 (13)	0.0295 (12)	0.0024 (9)	−0.0051 (9)	0.0019 (10)
C4B	0.0300 (12)	0.0246 (11)	0.0281 (11)	0.0028 (10)	−0.0030 (10)	0.0016 (9)
C5B	0.0277 (11)	0.0208 (11)	0.0268 (11)	−0.0004 (9)	−0.0017 (9)	−0.0001 (9)
C6B	0.0436 (15)	0.0250 (12)	0.0490 (16)	0.0054 (12)	−0.0147 (13)	0.0054 (12)
O1B	0.0288 (8)	0.0233 (8)	0.0378 (10)	0.0010 (7)	−0.0088 (8)	−0.0037 (7)
O2B	0.0280 (8)	0.0267 (9)	0.0382 (10)	−0.0038 (7)	−0.0098 (8)	0.0084 (8)
N3B	0.0256 (10)	0.0254 (10)	0.0327 (10)	−0.0018 (8)	−0.0074 (8)	0.0038 (8)
C7B	0.0348 (13)	0.0256 (13)	0.0470 (15)	−0.0031 (11)	−0.0183 (12)	0.0069 (11)
C8B	0.0367 (13)	0.0322 (14)	0.0432 (15)	−0.0102 (12)	−0.0176 (12)	0.0065 (12)
C9B	0.0376 (14)	0.0236 (12)	0.0385 (14)	−0.0115 (10)	−0.0126 (12)	0.0055 (11)
C10B	0.0294 (12)	0.0200 (11)	0.0320 (12)	−0.0043 (9)	−0.0048 (10)	0.0048 (9)
C11B	0.0207 (10)	0.0246 (11)	0.0227 (10)	−0.0032 (9)	0.0004 (8)	0.0026 (9)
C12B	0.0206 (9)	0.0237 (11)	0.0222 (10)	−0.0007 (9)	0.0007 (8)	0.0016 (9)
O1W	0.018 (3)	0.017 (3)	0.032 (3)	0.000 (2)	0.005 (3)	−0.001 (3)
O2W	0.122 (3)	0.0424 (13)	0.0626 (15)	−0.0350 (16)	0.0451 (17)	−0.0191 (12)

### Geometric parameters (Å, °)

**Table:**

N1A—C1A	1.347 (3)	N1B—H1NB	0.95 (4)
N1A—C5A	1.365 (3)	N2B—C1B	1.329 (3)
N1A—H1NA	0.97 (4)	N2B—H2NB	0.82 (3)
N2A—C1A	1.331 (3)	N2B—H3NB	0.93 (3)
N2A—H2NA	0.96 (4)	C1B—C2B	1.423 (3)
N2A—H3NA	0.94 (3)	C2B—C3B	1.353 (3)
C1A—C2A	1.423 (3)	C2B—H2BA	0.9300
C2A—C3A	1.368 (3)	C3B—C4B	1.421 (4)
C2A—H2AA	0.9300	C3B—H3BA	0.9300
C3A—C4A	1.412 (3)	C4B—C5B	1.371 (3)
C3A—H3AA	0.9300	C4B—C6B	1.491 (3)
C4A—C5A	1.368 (3)	C5B—H5BA	0.9300
C4A—C6A	1.502 (3)	C6B—H6BA	0.9600
C5A—H5AA	0.9300	C6B—H6BB	0.9600
C6A—H6AA	0.9600	C6B—H6BC	0.9600
C6A—H6AB	0.9600	O1B—C12B	1.241 (3)
C6A—H6AC	0.9600	O2B—C12B	1.262 (3)
O1A—C12A	1.223 (3)	N3B—C7B	1.340 (3)
O2A—C12A	1.273 (3)	N3B—C11B	1.350 (3)
N3A—C11A	1.333 (3)	C7B—C8B	1.391 (4)
N3A—C7A	1.343 (3)	C7B—H7BA	0.9300
C7A—C8A	1.382 (4)	C8B—C9B	1.372 (4)
C7A—H7AA	0.9300	C8B—H8BA	0.9300
C8A—C9A	1.376 (4)	C9B—C10B	1.389 (3)
C8A—H8AA	0.9300	C9B—H9BA	0.9300
C9A—C10A	1.395 (3)	C10B—C11B	1.384 (3)
C9A—H9AA	0.9300	C10B—H10B	0.9300
C10A—C11A	1.393 (3)	C11B—C12B	1.524 (3)
C10A—H10A	0.9300	O1W—H1W1	0.8098
C11A—C12A	1.523 (3)	O1W—H2W1	0.8148
N1B—C1B	1.353 (3)	O2W—H1W2	0.8172
N1B—C5B	1.360 (3)	O2W—H2W2	0.8226
			
C1A—N1A—C5A	123.2 (2)	C1B—N1B—H1NB	123 (2)
C1A—N1A—H1NA	114 (2)	C5B—N1B—H1NB	114 (2)
C5A—N1A—H1NA	122 (2)	C1B—N2B—H2NB	117 (2)
C1A—N2A—H2NA	118 (2)	C1B—N2B—H3NB	117 (2)
C1A—N2A—H3NA	123.3 (19)	H2NB—N2B—H3NB	126 (3)
H2NA—N2A—H3NA	119 (3)	N2B—C1B—N1B	119.1 (2)
N2A—C1A—N1A	119.2 (2)	N2B—C1B—C2B	123.9 (2)
N2A—C1A—C2A	123.5 (2)	N1B—C1B—C2B	117.0 (2)
N1A—C1A—C2A	117.2 (2)	C3B—C2B—C1B	119.9 (2)
C3A—C2A—C1A	120.0 (2)	C3B—C2B—H2BA	120.0
C3A—C2A—H2AA	120.0	C1B—C2B—H2BA	120.0
C1A—C2A—H2AA	120.0	C2B—C3B—C4B	122.2 (2)
C2A—C3A—C4A	121.1 (2)	C2B—C3B—H3BA	118.9
C2A—C3A—H3AA	119.4	C4B—C3B—H3BA	118.9
C4A—C3A—H3AA	119.4	C5B—C4B—C3B	116.0 (2)
C5A—C4A—C3A	117.3 (2)	C5B—C4B—C6B	121.5 (2)
C5A—C4A—C6A	121.2 (2)	C3B—C4B—C6B	122.6 (2)
C3A—C4A—C6A	121.5 (2)	N1B—C5B—C4B	121.8 (2)
N1A—C5A—C4A	121.2 (2)	N1B—C5B—H5BA	119.1
N1A—C5A—H5AA	119.4	C4B—C5B—H5BA	119.1
C4A—C5A—H5AA	119.4	C4B—C6B—H6BA	109.5
C4A—C6A—H6AA	109.5	C4B—C6B—H6BB	109.5
C4A—C6A—H6AB	109.5	H6BA—C6B—H6BB	109.5
H6AA—C6A—H6AB	109.5	C4B—C6B—H6BC	109.5
C4A—C6A—H6AC	109.5	H6BA—C6B—H6BC	109.5
H6AA—C6A—H6AC	109.5	H6BB—C6B—H6BC	109.5
H6AB—C6A—H6AC	109.5	C7B—N3B—C11B	116.8 (2)
C11A—N3A—C7A	117.5 (2)	N3B—C7B—C8B	123.8 (2)
N3A—C7A—C8A	124.0 (3)	N3B—C7B—H7BA	118.1
N3A—C7A—H7AA	118.0	C8B—C7B—H7BA	118.1
C8A—C7A—H7AA	118.0	C9B—C8B—C7B	118.7 (2)
C9A—C8A—C7A	118.1 (2)	C9B—C8B—H8BA	120.7
C9A—C8A—H8AA	120.9	C7B—C8B—H8BA	120.7
C7A—C8A—H8AA	120.9	C8B—C9B—C10B	118.6 (2)
C8A—C9A—C10A	119.0 (2)	C8B—C9B—H9BA	120.7
C8A—C9A—H9AA	120.5	C10B—C9B—H9BA	120.7
C10A—C9A—H9AA	120.5	C11B—C10B—C9B	119.3 (2)
C11A—C10A—C9A	118.8 (2)	C11B—C10B—H10B	120.3
C11A—C10A—H10A	120.6	C9B—C10B—H10B	120.3
C9A—C10A—H10A	120.6	N3B—C11B—C10B	122.8 (2)
N3A—C11A—C10A	122.6 (2)	N3B—C11B—C12B	116.7 (2)
N3A—C11A—C12A	116.7 (2)	C10B—C11B—C12B	120.5 (2)
C10A—C11A—C12A	120.7 (2)	O1B—C12B—O2B	126.5 (2)
O1A—C12A—O2A	125.7 (2)	O1B—C12B—C11B	117.7 (2)
O1A—C12A—C11A	118.3 (2)	O2B—C12B—C11B	115.8 (2)
O2A—C12A—C11A	116.0 (2)	H1W1—O1W—H2W1	114.2
C1B—N1B—C5B	123.0 (2)	H1W2—O2W—H2W2	111.4
			
C5A—N1A—C1A—N2A	−179.8 (2)	C5B—N1B—C1B—N2B	179.2 (2)
C5A—N1A—C1A—C2A	0.8 (3)	C5B—N1B—C1B—C2B	0.1 (3)
N2A—C1A—C2A—C3A	−179.4 (2)	N2B—C1B—C2B—C3B	−179.2 (2)
N1A—C1A—C2A—C3A	0.0 (3)	N1B—C1B—C2B—C3B	−0.2 (3)
C1A—C2A—C3A—C4A	−0.6 (4)	C1B—C2B—C3B—C4B	0.2 (4)
C2A—C3A—C4A—C5A	0.4 (3)	C2B—C3B—C4B—C5B	−0.1 (4)
C2A—C3A—C4A—C6A	−177.5 (2)	C2B—C3B—C4B—C6B	−179.6 (3)
C1A—N1A—C5A—C4A	−1.0 (3)	C1B—N1B—C5B—C4B	0.0 (4)
C3A—C4A—C5A—N1A	0.4 (3)	C3B—C4B—C5B—N1B	0.0 (4)
C6A—C4A—C5A—N1A	178.3 (2)	C6B—C4B—C5B—N1B	179.5 (2)
C11A—N3A—C7A—C8A	1.3 (5)	C11B—N3B—C7B—C8B	0.0 (4)
N3A—C7A—C8A—C9A	−0.3 (5)	N3B—C7B—C8B—C9B	−1.1 (5)
C7A—C8A—C9A—C10A	−0.6 (4)	C7B—C8B—C9B—C10B	0.6 (4)
C8A—C9A—C10A—C11A	0.6 (4)	C8B—C9B—C10B—C11B	0.9 (4)
C7A—N3A—C11A—C10A	−1.4 (4)	C7B—N3B—C11B—C10B	1.6 (4)
C7A—N3A—C11A—C12A	177.5 (3)	C7B—N3B—C11B—C12B	−177.3 (2)
C9A—C10A—C11A—N3A	0.5 (4)	C9B—C10B—C11B—N3B	−2.1 (4)
C9A—C10A—C11A—C12A	−178.3 (2)	C9B—C10B—C11B—C12B	176.8 (2)
N3A—C11A—C12A—O1A	11.8 (4)	N3B—C11B—C12B—O1B	4.8 (3)
C10A—C11A—C12A—O1A	−169.3 (3)	C10B—C11B—C12B—O1B	−174.2 (2)
N3A—C11A—C12A—O2A	−166.9 (3)	N3B—C11B—C12B—O2B	−175.6 (2)
C10A—C11A—C12A—O2A	12.0 (4)	C10B—C11B—C12B—O2B	5.4 (3)

### Hydrogen-bond geometry (Å, °)

**Table:**

*D*—H···*A*	*D*—H	H···*A*	*D*···*A*	*D*—H···*A*
O2W—H2W2···O2A^i^	0.82	2.00	2.812 (2)	170
N1A—H1NA···O2B^ii^	0.99 (2)	1.69 (2)	2.669 (2)	170 (2)
N2A—H2NA···O1B^ii^	0.94 (3)	1.89 (3)	2.829 (2)	178 (3)
N2A—H3NA···N3A	0.94 (3)	2.08 (3)	3.019 (3)	177 (2)
N1B—H1NB···O2A^i^	0.96 (3)	1.68 (3)	2.642 (2)	173 (3)
N2B—H2NB···N3B	0.83 (3)	2.22 (3)	3.040 (2)	173 (2)
N2B—H3NB···O1A^i^	0.93 (2)	1.90 (2)	2.831 (3)	175 (2)
C5A—H5AA···O2W^iii^	0.93	2.39	3.319 (3)	175
C7A—H7AA···O2B^iv^	0.93	2.42	3.217 (3)	144
C8A—H8AA···O2W^v^	0.93	2.50	3.339 (3)	151

Symmetry codes: (i) *x*−1, *y*, *z*; (ii) *x*+1, *y*, *z*; (iii) *x*+1, *y*+1, *z*; (iv) *x*+1/2, −*y*+3/2, −*z*; (v) *x*+1/2, −*y*+1/2, −*z*.
